# Evaluation of microplastics isolated from sea cucumber A*caudina molpadioides* in Pulau Langkawi, Malaysia

**DOI:** 10.1016/j.heliyon.2023.e16822

**Published:** 2023-05-30

**Authors:** Nurzafirah Mazlan, Sarah Syazwani Shukhairi, Miftahul Jannah Muhammad Husin, Jemimah Shalom, Safaa Najah Saud, Muhammad Shirwan Abdullah Sani, Meng Chuan Ong, Naveen Kumar Naidu Chandra Mohan, Nor Asyikin Sopian

**Affiliations:** a Borneo Marine Research Institute, Universiti Malaysia Sabah, Jalan UMS, 88400 Kota Kinabalu, Sabah, Malaysia; b Faculty of Health & Life Sciences, Management and Science University (MSU), 40100 Shah Alam, Selangor, Malaysia; c Faculty of Science and Marine Environment, University Malaysia Terengganu, 21300 Kuala Nerus, Terengganu, Malaysia; d Faculty of Information Sciences and Engineering, Management and Science University (MSU), 40100 Shah Alam, Selangor, Malaysia; e International Institute for Halal Research and Training, Level 3, KICT Building, International Islamic University Malaysia, Jalan Gombak, Kuala Lumpur, Malaysia; f Faculty of Industrial Sciences and Technology, Universiti Malaysia Pahang, 26300 Gambang, Kuantan, Malaysia

**Keywords:** Microplastic, Sea cucumber, Seafood, FTIR analysis

## Abstract

Plastic pollution is an emerging environmental concern in recent years due to continuous mass production and its slow degradation. Microplastics measuring between 5 mm and 1 μm are being ingested by marine animals and eventually by human consumption in form of seafood. The aim of this research was to evaluate microplastics isolated from sea cucumber *Acaudina molpadioides* in Pulau Langkawi. A total of 20 animals were collected and their gastrointestinal tract were digested using NaOH. Microplastics were isolated, filtered and identified through microscopic examination based on the colour, shape and size. The chemical composition of microplastics were further analyzed by FTIR to identify the functional group of polymers. A total of 1652 microplastics were found in *A. molpadioides.* Fibres (99.4%) and black color (54.4%) were the majority of microplastics observed in terms of shapes and colors. The size range within 0.5–1 μm and 1–2 μm were the highest abundance observed. There were two identified polymer types of microplastics obtained through FTIR which were polyethylene (PE) and polymethyl methacrylate (PMMA). In conclusion, microplastics were isolated from the gastrointestinal tract of *A. molpadioides* indicating that the animals were contaminated. Further research can be done on the toxicity effects of these microplastics towards human upon consumption of these animals as seafood.

## 1 Introduction

Malaysia is known to be famous with their beaches and islands as well as their cultural diversity and mouth-watering cuisines that are rich with health benefits. Exotic seafood famous among local communities at Pulau Langkawi for its significant bioactive compound and potential therapeutic effects in human health and well-being, *A. molpadioides* or known as ‘beronok’ locally are widely distributed at sandy bottoms of the island [33]. It can be eaten raw when young or even cooked in various cuisines such as kerabu beronok (salad) or soup. *A. molpadioides* not only rich with nutritious values but also plays a crucial role in improving the water quality by acting as a bioindicator where it filters organic matter and consume tiny particles from the marine environment.

Plastics are made up of wide range of materials with characteristics of being lightweight, resilience, corrosion resistance, transparency and easy production, making it ideal for various applications in our life [34]. However, due to high demands and disposal, plastic production is expected to have tripled and will account for a fifth of global oil consumption by 2050 causing serious environmental pollution and management problems [14, 29]. Global plastic demands are dominated by thermoplastic polymer types of polypropylenes, polyethylene, polyvinyl chloride, polystyrene, polymethyl methacrylate and others.

Microplastics pollution and its effects have caught the eye of public in recent years due to the high potential of microplastics accumulating in the food chain. Microplastics were described as the breaking down of plastic items into smaller pieces below than 5 mm in diameter where it can release in the form of tiny particles directly into the environment or produced by weathering of larger plastics through changing of environmental conditions such as the exposure of wave action and wind abrasion [13,31]. These tiny plastic polymers can be ingested and accumulate within the marine animals such as their tissues, circulatory system and even brain as they are likely mistaking it as food. With the consumption of a large amount of toxic chemical contained in plastic production including polyethylene terephthalate (PET), bisphenol A (BPA) and pesticides has leads to various health threats such as decreased functionality of organs, development of biological effects and even increased mortality in marine animals [4]; Carrington, 2020; [32,38].

Due to its effective uptake and assimilation of particles, the small size of microplastics gives the potential of ingestion, which has been demonstrated in a number of marine animals [5,12]; Maciej [21,27,37]. With rapidly increased production and purchasing of marine products these days, higher global per capita seafood consumption has been recorded [2]. Therefore, consuming seafood are one way for humans to be exposed to microplastics ingestion. Research data on microplastics and the presence of microplastics in benthic filter feeders specifically sea cucumbers towards human health upon consumption as seafood have been studied comparably less than other marine species due to inadequate evidence to perform a risk assessment. Thus, it is important to evaluate the type of microplastics and the potential health threats of humans, including dietary exposure in order to assess the contributing risk of contaminated seafood.

## 2 Materials and methods

### 2.1 Sample collection & preparation

Twenty samples of *A. molpadioides* were collected along the beach of Kuala Muda, Pulau Langkawi. All samples were transported immediately to the laboratory of Management and Science University after sample collection for further analysis of microplastics. *A. molpadioides* samples were stored in clean glass bottle at −20 °C. Measurement of body weight and length of *A. molpadioides* was recorded prior to removal of gastrointestinal tract. The dissected samples were stored in a glass bottle containing 95% ethanol solution until further analysis [25].

### 2.2 Isolation of microplastic from gastrointestinal tract of *A. molpadioides*

The tissue digestion was done according to Ref. [18]. The isolated tissue was placed in a 100 ml glass beaker with 50 ml of 10% sodium hydroxide (NaOH) to digest the soft tissue following 2 days of incubation at 40 °C. After digestion, the digestates were filtered through micro-glass fibers filter paper (pore size 5–13 μm) using a vacuum system. For isolation of high-density particles, filter paper was soaked in concentrated salt solution such as 4.4 M sodium iodide (Nal), sonicated at 50 Hz for 5 min and agitated on orbital shaker at 200 rpm for 5 min. The solution was then centrifuged at 500×*g* for 2 min [11]. The solution was carefully transferred and filtered through 1.2 μm glass micro-glass fibre filter papers under vacuum filtration. The filter was placed in a new covered glass Petri dish to avoid contamination during storage and air dried at room temperature for 24 h as wet filters reflect the light of the microscope. The sonication, agitation and filtration steps were repeated twice to ensure the maximum recovery of microplastics [11].

### 2.3 Microscopic examination of microplastics

Isolation of microplastics on the filter papers was carefully observed horizontally and vertically under a dissecting microscope and photographed using comparison microscope. Microplastics were sorted out and recorded on a data sheet based on its size, shape and colour. Hot needle test method was used in cases of unable to differentiate between microplastics and organic materials. The collected microplastics were placed onto a Petri dish with filter paper for identification of polymer chemical properties [25].

### 2.4 Identification on the polymer type of microplastics by fourier transform infrared spectroscopy (FTIR) analysis

The FTIR analysis to identify plastics components by characterising the molecular markup of polymers was performed using Thermo Fisher Scientific NICOLET iS50 FTIR Spectrometer. The instrument was equipped with deuterated triglycine sulfate (DTGS)-potassium bromide (KBr) detector and XT-KBR beam splitter. A background scan was collected before each sample on a clean tape and recorded as blank using attenuated total reflection (ATR) imaging, using 32 co-added scans in the mid-IR range of 4000–650 cm^−1^. The sample was placed and in direct contact with the diamond surface with ATR imaging using a pressure probe during data acquisition with the same settings as for the background scan. The blank spectrum was deducted in order to obtain the actual spectrum of microplastics. Identification on the type of microplastics were analyzed based on FTIR spectrum range of previous studies [16,26].

### 2.5 Quality assurance & quality control

Non plastics clothes such as cotton lab coat and nitrile/latex gloves were worn throughout the research. Lab instruments such as glassware, and metal tools were used to analyse the samples. During filtration and sieving, any possible contamination was checked. Air exposure should be minimized to avoid any loss of microplastics.

## 3 Results and discussion

### 3.1 Characteristics of microplastics

A total of 20 samples of *A. molpadioides* were studied. Their gastrointestinal tracts were found contaminated with microplastics. The abundance of microplastics ranged from 0.1 to 6.0 μm in length with an average length of 0.5–1 μm. A total number of 1652 microplastics were isolated from 20 samples of the gastrointestinal tract of *A. molpadioides* with an average of 83 microplastics per sea cucumber.

**Fig. 1:**
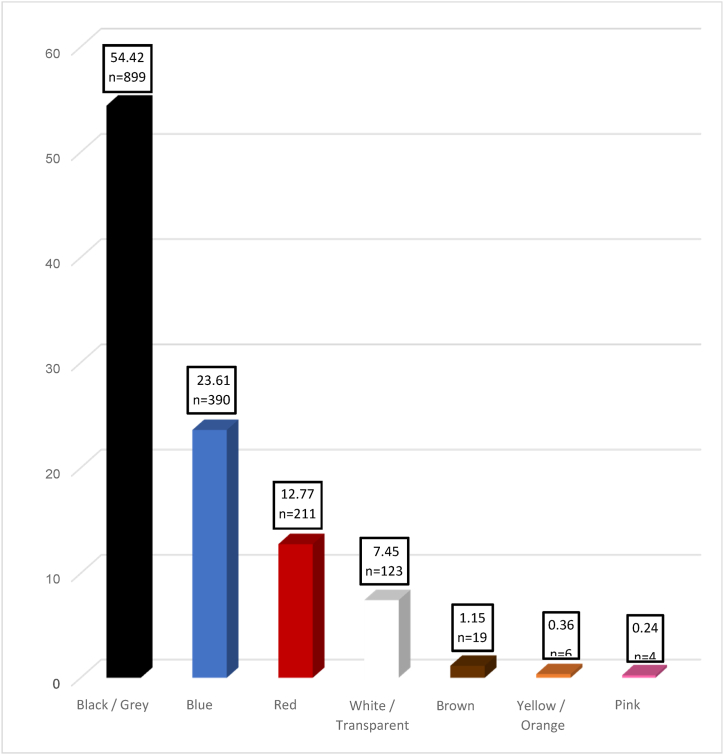
Percentage abundance (%) of microplastics isolated from gastrointestinal tract of *Acaudina molpadioides* based on its colors.

Fig. 1 shows the abundance of microplastics isolated from gastrointestinal tract of *A. molpadioides* based on its colors. Particles of microplastics that were found were eight different colors which includes black, blue, brown, pink, red, yellow, orange and white. Black colored particles were the most dominant with 54.42% for *A. molpadioides*. Black microplastics followed by blue (23.61%), red (12.77%), white (7.45%), brown (1.15%), yellow/orange (0.36%) and pink (0.24%) were also observed for *A. molpadioides*. The primary source determines the colour of microplastics although it can be affected by UV radiation, weathering and microbial deterioration [39]. Microplastic colors observed in the present study was similar to those studies recorded previously where black and blue are known to be notable in commercial fishing industry such as fishing nets and ground ropes [24].

**Fig. 2:**
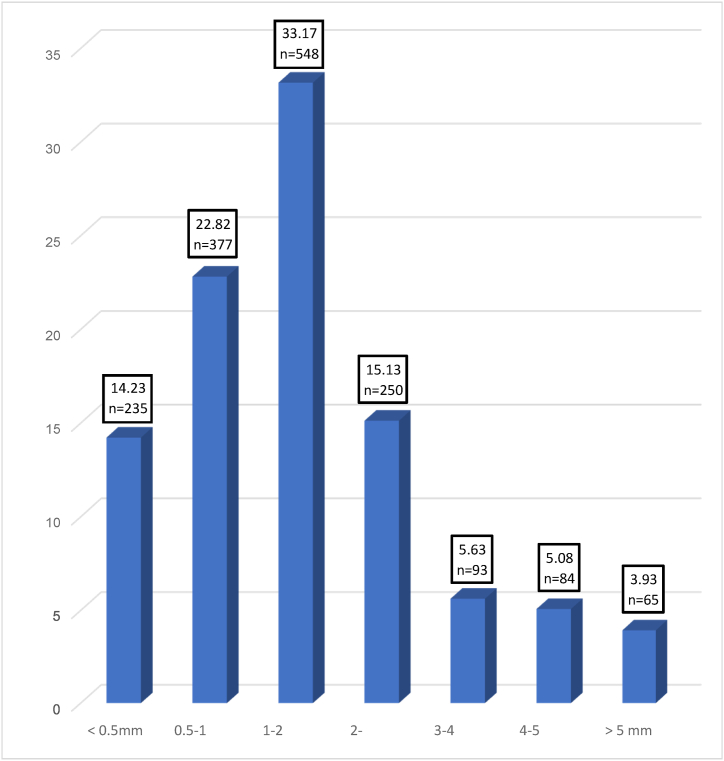
Percentag abundance (%) of microplastics isolated from gastrointestinal tract of *Acaudina molpadioides* based on its size.

There is no uniform standard on measuring microplastics and observed microplastic sizes in this study range from lesser than 0.5 mm to more than 5 mm in length. The size range of microplastics is important because it defines the pollutants’ potential adverse effect on ecosystem biota. Based on the result obtained in Fig. 2, the size range within 0.5–1 mm and 1–2 mm were the highest abundance class size for *A. molpadioides*. *A. molpadioides* showed 22.82% and 33.17% for both sizes, respectively. Microplastics that are larger than 5 mm shows 3.93%. The physicochemical characteristics of plastics will undergo significant changes as soon as they enter the aquatic system due to the rapid development of microbial film [20,30]. Plastics that were previously buoyant may sink into the water column as a result of the development of biofilm. With progressive degradation, these fragmented microplastics will continue to produce smaller size particles in a large quantity and sink over time, which eventually become accessible to the ocean’s benthic zone. Benthic filter feeder such as sea cucumber where they adopt non selective feeding strategy where huge amount of sediment are ingested making them vulnerable to sinking microplastics particles.

**Fig. 3:**
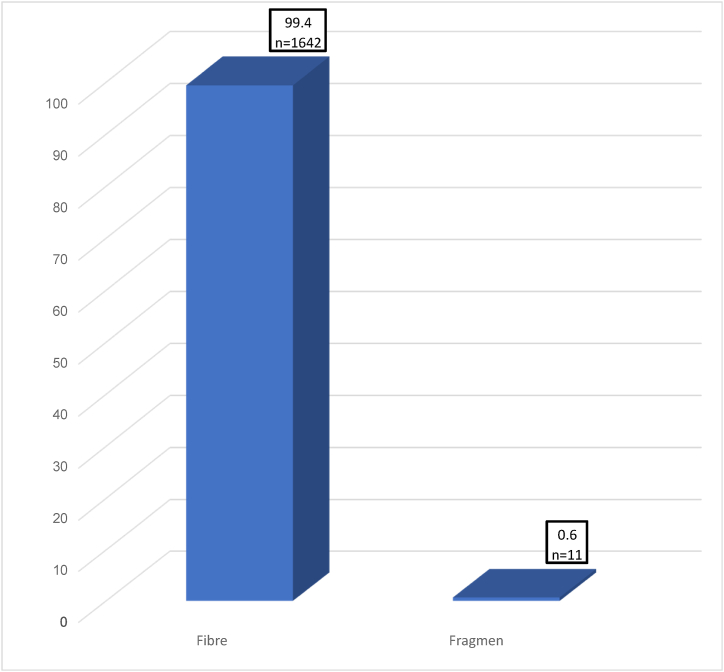
Percentage abundance (%) of microplastics isolated from gastrointestinal tract of *Acaudina molpadiodes* based on its shapes.

**Fig. 4:**
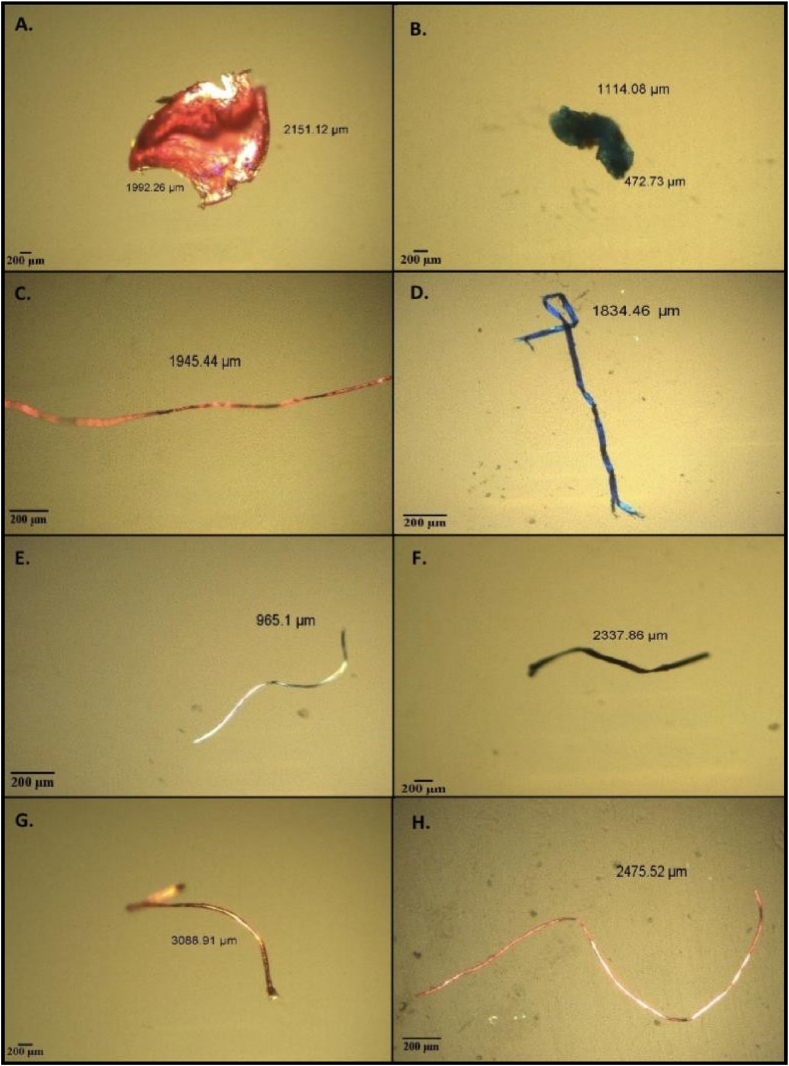
The shapes and colors of microplastic isolated from gastrointestinal tract (GIT) of sea cucumber, *Acaudina molpadiodes.* A) Red fragment, B) Blue fragment, C) Pink fibre, D) Blue fibre, E) White fibre, F) Black fibre, G) Brown fibre, and H) Pink fibre [Scale bars: 200 μm]

Fibres were the most microplastics found in this study followed by fragments as shown in Fig. 3. Fibres were the greatest type of microplastics that were accounted for 99.4%. The proportion of fragments was low but were observed in this species. Fragmented shape of microplastics in *A. molpadioides* were accounted at 0.6%. This is similar to other research studies that reported fibers were the most prevalent type of microplastic found in all studies [8,11,19,28]. Various colors of microplastics were also isolated from the samples such as red, blue, pink, white and red (Fig. 4). The environmental dispersion of these plastic particles has been extensively researched. The microplastics are most likely secondary in nature derived from degradation of larger plastic materials into smaller pieces as they were driven by environmental processes for a long period of time such as high temperature, oxidative weathering and ultraviolet radiation [3]. These factors eventually cause embrittlement that encourages fracturing, leading it to become vulnerable to physical abrasion and fragmentation [9,36]. Studies also have proven that sewage polluted by fibres from washing clothes tends to be a major source of microplastics because high representation of acrylic microfibres having lower tenacity and easy to break as they are least favorable to trap in washing machine [6,7].

### 3.2 Chemical composition of microplastics

**Fig. 5:**
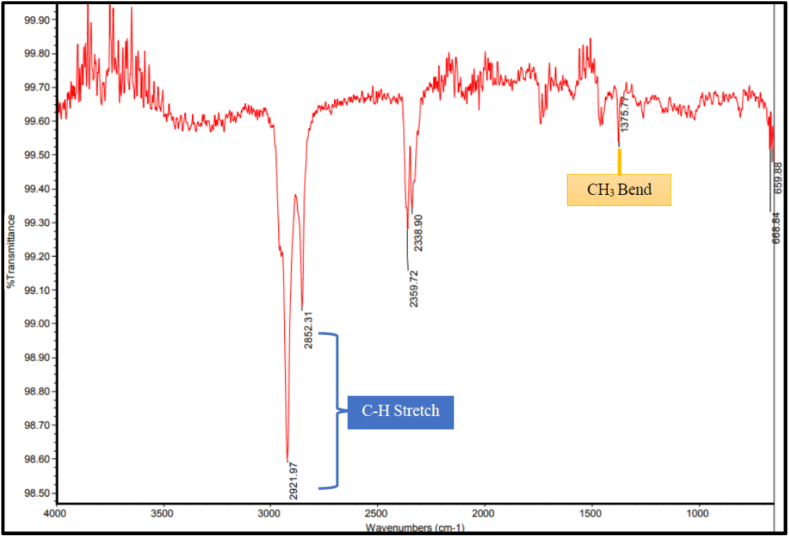
FTIR spectrum showing the functional group of polyethylene composition obtained from gastrointestinal tract of *Acaudina molpadiodes*.

**Fig. 6:**
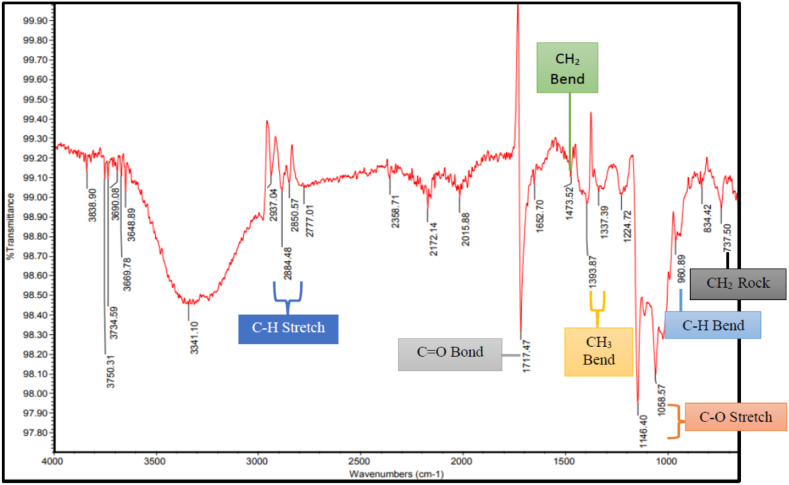
FTIR spectrum showing the functional group of polymethyl methacrylate composition obtained from gastrointestinal tract of *Acaudina molpadiodes*.

From FTIR analysis of the most prominent microplastic particles, the polymers identified in *A. molpadioides* were derived from polyethylene and polymethyl methacrylate. The FTIR spectra associated to microplastic as examples are shown in Fig. 5, Fig. 6.

In Fig. 5, the FTIR spectrum obtained shows the absorption band of aromatic C–H stretching vibration recorded around 2852.31 cm^−1^ to 2921.97 cm^−1^. The absorption band of CH_3_ bend observed at peak with wavelength of 1375.77 cm^−1^. Both FTIR polyethylene spectra carrying major peaks linked to alkyl functional group and was a close agreement as reported from previous studies on the identification of commercial polymers contained in plastic ingested by endangered marine animals and environment [16]. Higher peaks and broader bands could indicate that the polymer has been oxidized and chemically altered depending on the length of time taken for the sample to exposed to the UV radiation [17]. Polyethylene is classified mainly into three types where branching affects the crystallinity that are based on density including low-density polyethylene (LDPE), linear low-density polyethylene (LLDPE) and high-density polyethylene (HDPE). According to Ref. [29]; polyethylene is one of the most produced thermoplastic polymers worldwide which float in marine waters. Some of the major application of polyethylene include general packaging such as food packaging, containers, pipes, tubes and other household items. Plastic carrier bags, which are often made of polyethylene, account for a significant amount of plastic pollution. Other than that, commercial fishing gear made of polyethylene could contribute the most microplastics because of mechanical abrasion against seabed [2]. Since Pulau Langkawi are known as tourist attraction famous for its leisure activities, this polymer is not surprisingly found as there is improper disposal of this polymer products in the marine ecosystem. With the effects of environmental factors such as mechanical stress and radiation, it leads to fragmentation into secondary microplastics over long period of time.

All major peaks shown in Fig. 6 were made up of carboxylic ester functional group and were found similar to the spectra reported by Refs. [16,35] on polymethyl methacrylate (PMMA). The strong C–H stretch absorption band can be seen around at peak of 2777.01 cm^−1^ to 2937.04 cm^−1^. The presence of carbonyl compound stretching (C=O) which are associated with PMMA polymer shows intense absorption bands at peaks of 1717.47 cm^−1^ while the peaks of CH_2_ bending were observed at 1473.52 cm^−1^. The peak of 1337.39 cm^−1^ and 1393.87 cm^−1^ observed attributed to the CH_3_ bend. Medium bands at 1058.57 cm^−1^ to 1146.40 cm^−1^ are assigned to C–O bond stretching modes of PMMA at fingerprint region where the pattern of peaks is complicated as each different compound produces its own unique pattern. C–H bend was shown at peak 960.89 cm^−1^. Other absorption band that was observed in Figure showed CH_2_ rock at a peak of 737.50 cm^−1^. Due to its high impact strength and shatter resistance, PMMA are widely used in replacement for inorganic glass making it as a first choice for automotive industries and marine transportation industries. Pulau Langkawi are known to be one of the most important shipping lanes in the world, thus it can caused microplastics pollution in the coastal water of Peninsular Malaysia from sea-based activities including shipping activities and aquaculture. With the weathering of PMMA’s by-products such as window pieces coming from ship, yacht and boat in salt water and thermal oxidative degradation over a period of time, it becomes brittle and crack [22]. Since PMMA has greater density, it will be carried by water currents then sink to the sea floor and consumed by filter feeders such as bivalves and echinoderms, eventually causing obstruction in the gastrointestinal tract of the marine organisms.

**Fig. 7:**
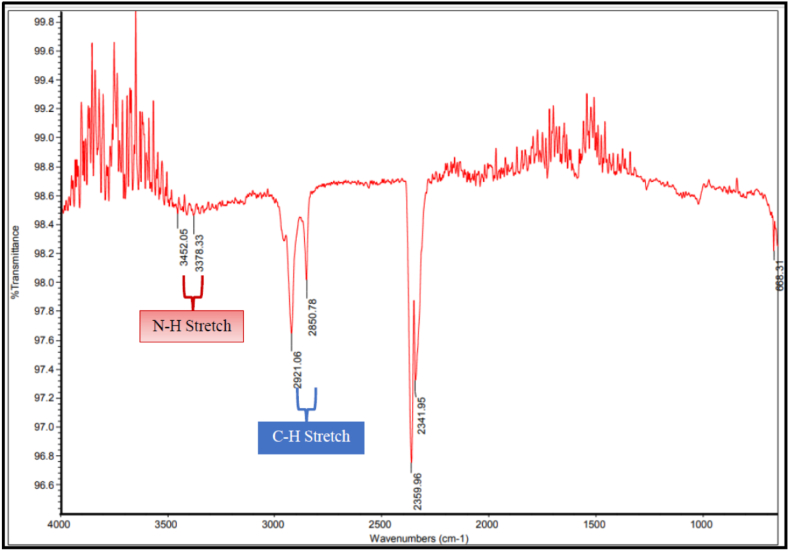
FTIR spectrum showing the unidentified functional group obtained from gastrointestinal tract of Acaudina molpadiodes.

Fig. 7 were shown as unidentified plastic particles as the peak of the functional groups were not significant as they were unrelated to any forms of polymers which were studied previously. The microplastics could be a mistake for its organic debris and other spherical anthropogenic particles such as algae, fish scales, glass, metal fume, particles in road paint, ceramic flakes etc. Furthermore, natural materials like cotton, linen and wool as well as semi synthetic polymers such as rayon can be included due to airborne fibre contamination [9,23].

With an increasing demand of seafood consumption, more research on microplastics in marine species should be done since there is still lacking research data on microplastics. The risk assessment of microplastics in seafood upon consumption are still being assessed and have yet been fully established. There are very few reports available on the monitoring of microplastics in food and its impact on human since the findings such as toxicological data in determining a potential health risk upon consumption of seafood species are unclear and rather controversial. Quality control and quality assurance were practiced to minimize contamination and avoid sample lost, however, procedural blanks were not used and thus the results could be speculated as overestimation of particles ingested by the sea cucumber. The missing of procedural blanks in methodology has also been reported in Refs. [18,25].

## 4 Conclusion

This study showed that microplastics were isolated and identified from *A. molpadiodes*. The main polymer types of microplastics identified in *A. molpadiodes* were polyethylene and polymethyl methacrylate. The abundance of microplastics detected were consistent with multiple previous studies. It is clear that humans are exposed to microplastics through seafood intake. With an increasing demand of seafood consumption, the result reported can be used to perform risk assessment for microplastics that affect the seafood species and human health. Further research can be done on the analysis and assessment of potential toxicology effects of these microplastics towards human health upon consumption of seafood species as the uncertainties remains regarding the extent of harm caused to marine species. The missing laboratory blank in the procedure could be the limitation in this study that may compromise with the actual number of particles ingested by the sea cucumber.

## Funding

The study was funded by Research Management Centre of 10.13039/501100015043Management and Science University (MSU) under Grant Agreement No SG-021-012019-FHLS.

## Author contribution statement

Sarah Syazwani Shukhairi: Miftahul Jannah Muhammad Husin: Performed the experiments; Analyzed and interpreted the data; Wrote the paper.

Nurzafirah Mazlan: Conceived and designed the experiments; Performed the experiments; Analyzed and interpreted the data; Contributed reagents, materials, analysis tools or data; Wrote the paper.

Jemimah Shalom: Naveen Kumar Naidu Chandra Mohan: Performed the experiments.

Safaa Najah Saud: Muhammad Shirwan Abdullah Sani: Analyzed and interpreted the data; Contributed reagents, materials, analysis tools or data.

Meng Chuan Ong: Nor Asyikin Sopian: Analyzed and interpreted the data.

## Data availability statement

Data will be made available on request.

## Declaration of competing interest

The authors declare that they have no known competing financial interests or personal relationships that could have appeared to influence the work reported in this paper.
